# Spent media analysis suggests cultivated meat media will require species and cell type optimization

**DOI:** 10.1038/s41538-022-00157-z

**Published:** 2022-09-29

**Authors:** Edward N. O’Neill, Joshua C. Ansel, Grace A. Kwong, Michael E. Plastino, Jenny Nelson, Keith Baar, David E. Block

**Affiliations:** 1 Department of Food Science and Technology, University of California, Davis, Davis, CA USA; 2 Department of Viticulture and Enology, University of California, Davis, Davis, CA USA; 3 Department of Chemical Engineering, University of California, Davis, Davis, CA USA; 4 Department of Molecular and Cellular Biology, University of California, Davis, Davis, CA USA; 5 Agilent Technologies, Santa Clara, CA USA; 6 Department of Neurobiology, Physiology, and Behavior, University of California, Davis, Davis, CA USA; 7 Department of Physiology and Membrane Biology, University of California, Davis, Davis, CA USA

**Keywords:** Biotechnology, Stem cells, Biomedical engineering, Chemical engineering

## Abstract

Cell culture media design is perhaps the most significant hurdle currently facing the commercialization of cultivated meat as an alternative source of dietary protein. Since media optimization for a specific culture system requires a significant amount of effort and investment, a major question remaining is whether media formulations can be easily shared across multiple production schemes for cells of different species and lineages. Here, we perform spent medium analysis to compare the specific nutrient utilization of primary embryonic chicken muscle precursor cells and fibroblasts to the murine C2C12 myoblast cell line. We demonstrate that these related cell types have significantly different nutrient utilization patterns collectively and on a per-cell basis, and that many components of conventional media do not appear to be depleted by the cells. Namely, glucose was not consumed as rapidly nor as completely by the chicken muscle precursors compared to other cells overall, and there were significant differences in specific consumption rates for several other key nutrients over the first day of culture. Ultimately, our results indicate that no one medium is likely ideal and cost effective to culture multiple cell types and that novel methods to streamline media optimization efforts will be important for the industry to develop.

## Introduction

Faced with a proliferation of climate-related disasters, zoonotic disease outbreaks, and human population growth in recent decades, society is now at a crossroads determining how to satisfy the ever-growing demand for quality dietary protein in a way that is responsible and sustainable^1^. Cultivated meat (CM) is gaining notable traction and investment in recent years as a potential solution to this issue^2,3^ and as a way to improve on the food safety, organoleptic, and nutritional attributes of conventional meat products^4–6^. However, presently, there are numerous technical hurdles to overcome before this food technology can be commercially viable at large scales^7^. As described in our recent review, the leading cost driver and challenge facing CM is the media used to culture the cells, since it is currently comprised of numerous indispensable and expensive components^8^.

In many avenues of published CM research, media is often taken for granted. Using traditional and commercially available biomedical cell culture media like Dulbecco’s Modified Eagle’s Medium (DMEM)—without regard to its cost or scalability—to develop cell lines and investigate the use of scaffolding for CM is common. Therefore, few advances have been made in the academic realm toward overcoming the myriad CM media challenges. While several academic researchers are now beginning to develop less expensive media that promotes better cell growth^9^, even with formulations that are animal product-free^10–12^, no studies have been performed to our knowledge that investigate the fundamental media requirements of CM-relevant cell types specifically. Compared to the standard approach today of trying to improve existing conventional biomedical media formulations, understanding these cells’ specific nutrient utilization rates will enable a much more directed approach to generating optimal media formulations for CM.

Spent media analysis (SMA) is a commonly used and fundamentally simple strategy for cell culture media optimization. Assuming one has the infrastructure, capabilities, or access to outside analytical services, SMA involves collecting samples of media and performing standard chemical analyses to measure the concentrations of important components and determine their rates of utilization. This approach is most suitable for understanding which media components are directly utilized by cells and should be supplied in greater quantities, those not consumed over time, and how waste products may accumulate with the potential to inhibit cell growth. SMA has been used successfully for a variety of industrial microbial and animal cell culture applications to reduce costs and improve scalability^13–15^; however, this approach has yet to be applied to CM media. SMA will have to be done in parallel with other approaches to media optimization since many components may be important to include in the media even if they are not consumed by the cells^16^.

In this study, we present the most comprehensive analysis that has been published to date on the metabolic nutrient utilization rates of three CM-relevant cell types. Primary embryonic chicken muscle precursors (cMPCs) and primary chicken muscle fibroblasts (cMFBs) were cultured alongside the commonly used murine C2C12 myoblast cell line in order to perform subsequent SMA to reveal species- and cell type-dependent variations in media requirements. Muscle precursor cells are characterized by their expression of early myogenesis-related genes as well as their capacity to differentiate and fuse into mature multinucleated muscle fibers; they are otherwise known as myoblasts in the embryo and satellite cells in the adult^8^. We decided to use chicken cells in this study due to the longstanding history of chicken primary cells as a model system in muscle physiology research and their relative ease of use^17^. Perhaps more importantly, there appears to be a general lack of information in the literature on cultivated chicken, despite conventional chicken being the fastest growing agricultural subsector today^18^. We were ultimately able to measure glucose, lactate, amino acids, water-soluble vitamins, trace elements and minerals, and certain growth factors in the spent media samples over the course of cell proliferation and differentiation in a small scale 2D multi-well plate format.

## Results

### Evaluating sugar utilization patterns during cell growth and differentiation

**Fig. 1:**
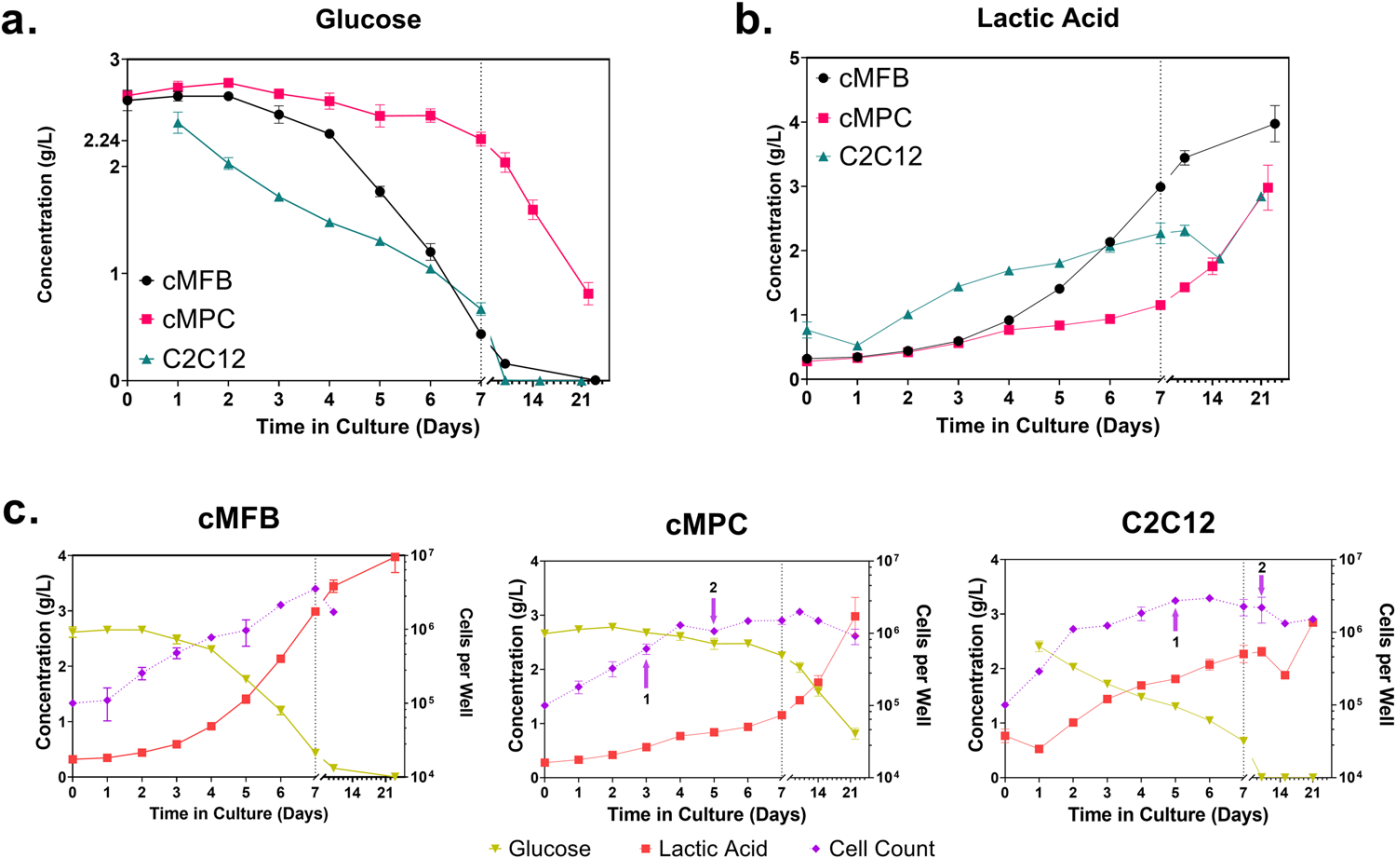
Glucose and LA + cell count. Analysis of sugars and organic acids in spent media from cultures of three different cultivated meat-relevant cell types. The three cell types were plated at 100,000 cells per well in 6-well plates, and the media from three wells per cell type were collected at each predetermined time point for chemical analysis. The concentrations of glucose (**a**) and lactic acid (**b**) in the spent media samples are plotted separately with each cell type’s trends overlaid. **c** The same data for the three cell types are each plotted separately with glucose and lactic acid concentrations overlaid. Cell counts per well in the 6-well plate experimental setup are overlaid in purple. Arrows pointing at cell count data points indicate differentiation status, with (1) indicating the time at which first signs of differentiation were apparent, and (2) indicating the time the maximum differentiation was reached. Data points represent the mean of three biological replicates ± standard deviation. cMFB, primary embryonic chicken muscle fibroblasts. cMPC, primary embryonic chicken muscle precursor cells. C2C12, murine myoblast-like cell line. The expected concentration of glucose in fresh media (40% high glucose DMEM, 40% Ham’s F10) is indicated in **a** at 2.24 g/L and is based on the manufacturer’s formulation sheet but does not include the contribution from the 20% fetal bovine serum in the experimental medium. Vertical dotted lines highlight the break in the *x*-axis scales at day 7 in **c**.

After all media samples were collected from all cell types and time points, the samples were analyzed via a high performance liquid chromatography (HPLC) method optimized for carbohydrate and organic acid analysis. The chromatogram peaks for glucose and lactic acid were the only two that displayed noticeable changes over the time course of spent media samples for any of the cell types. The integrated peak areas and heights of other distinguishable peaks in the chromatograms essentially remained constant across media samples for each cell type (Supplemental Fig. 1). Analysis of glucose concentration revealed a notable difference in the utilization patterns between the cMPCs and the other two cell types (Fig. 1a). The cMPCs exhibited a significantly lower rate of glucose utilization that appeared mostly linear over the observed culture period, while the cMFBs and C2C12s used glucose more rapidly and almost completely by D10, which also corresponds to the cell count plots leveling off. Lactate accumulated in the media following roughly the inverse path of glucose (Fig. 1b).

To assess how the cell types differed in their overall growth characteristics and help explain the patterns of nutrient utilization, we counted the cells that remained in the culture plates directly after collecting the spent media samples. All three cell types proliferated well throughout the observed culture period. The C2C12s grew exponentially for about 2 days after seeding (indicated by the roughly straight line on the semi-log cell count plot in Fig. 1c) and continued some growth for an additional 4 days. The cMFBs proliferated exponentially for 7 days, and the cMPCs for 4 days. While C2C12s appeared to proliferate slightly faster than the chicken cells during the first 5 days, all three cell types reached a fairly similar maximum cell concentration around 7 days, which corresponded to complete confluence in the 6-well plates (Fig. 1c). The cMPCs started differentiating into myotubes by day 3, even before the proliferation phase was complete, while the C2C12s started differentiating at day 5, around when the proliferation ended. The cMFBs did not display myotube formation at any point during the 3-week culture period. Overall, there was a gradual decline in the measurable cell numbers at the time points after 7 days. This could be attributable to cell death and/or fusion into multinucleated myotubes prior to cell counting.

### Assessment of nitrogen utilization during cell growth and differentiation

**Fig. 2:**
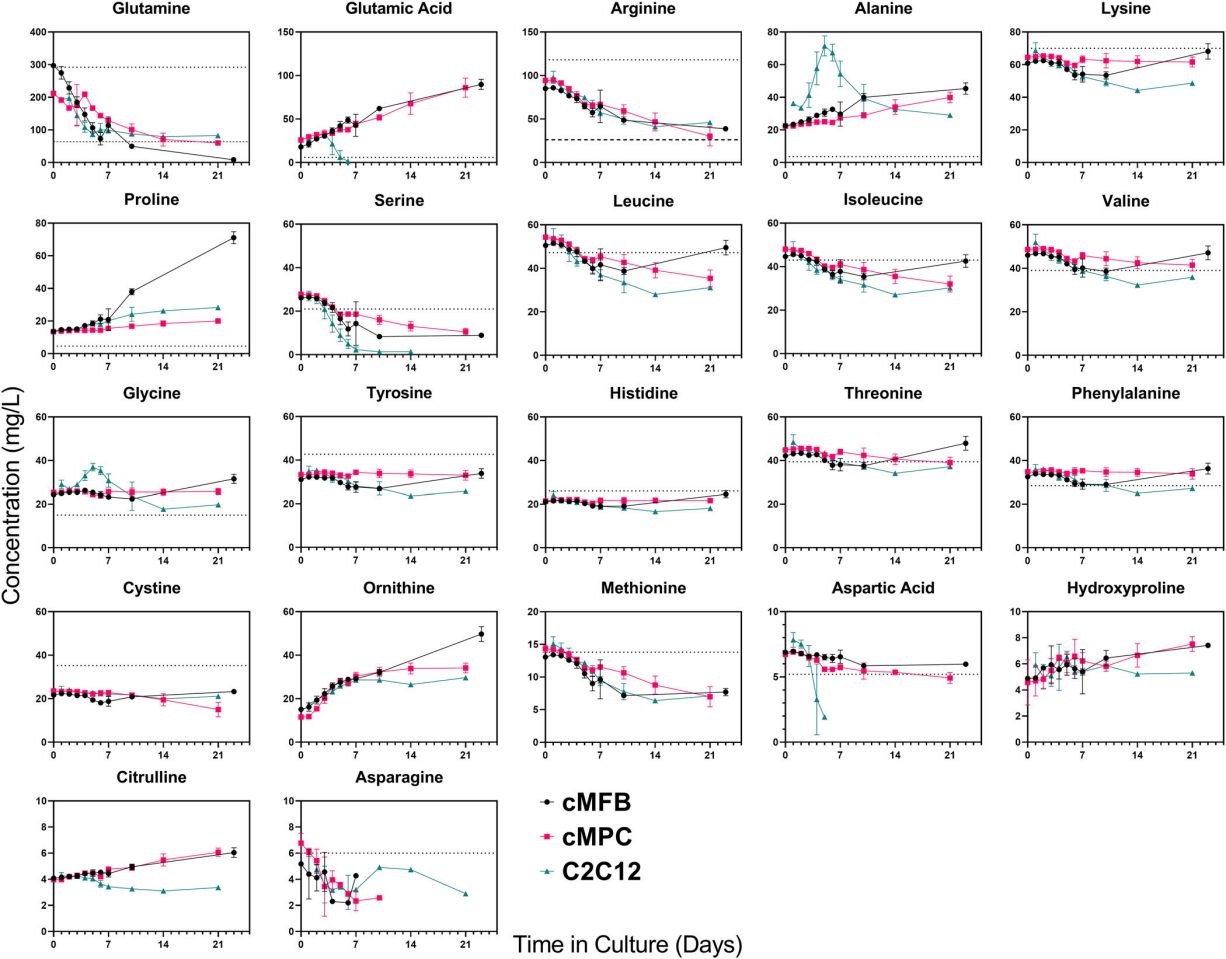
Amino acids. Analysis of amino acid concentrations in spent media from cultures of three cultivated meat-relevant cell types. Dotted lines represent expected concentrations of the amino acids based on the contributions from the basal media formulation consisting of 40% DMEM and 40% F10. The contribution from the 20% of fetal bovine serum in the media is not considered in the estimated expected starting concentrations. Data points represent the mean of three biological replicates ± standard deviation. The ranges of the *y*-axes for the graphs are not all the same. cMFB primary embryonic chicken muscle fibroblasts. cMPC primary embryonic chicken muscle precursor cells. C2C12 murine myoblast-like cell line.

To assess relative nitrogen utilization between the three cell types, we measured free amino acid concentrations in all of the spent media samples collected. This analysis was meant to evaluate the relative importance of specific amino acids in CM media. The data in Fig. 2 indicate that the concentrations of several amino acids do not significantly decrease in the media over time. Although, many other amino acids do in fact appear to be partially depleted by the cells—most notably four essential amino acids (arginine, isoleucine, leucine, and methionine) and two non-essential amino acids (glutamine and serine). On an absolute scale, glutamine is the amino acid used the most, followed by arginine and serine. The serine in the C2C12 culture was essentially the only amino acid that appeared to approach complete depletion—this occurred by day 7, which is also when the cell count began to slightly decrease (due to death and/or fusion). Other amino acids continued to be utilized after the end of exponential cell growth. Interestingly, proline in the media increased only in the cMFB culture after day 7. These data suggest that the cMFBs are differentiating and increasing the production and subsequent breakdown of collagen protein (roughly a fifth of all amino acids in collagen are proline). However, there was no significant difference in the consumption rates of any amino acids between the cell types during their proliferation phase.

### Vitamins and other micronutrients are not utilized significantly

**Fig. 3:**
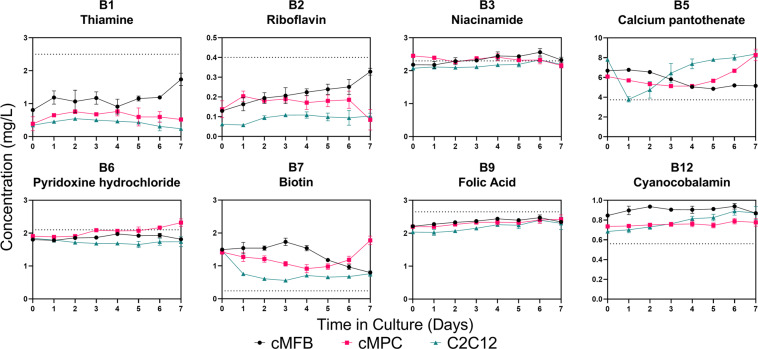
Water-soluble vitamins. Analysis of B-vitamin concentrations in spent media from cultures of three cultivated meat-relevant cell types, using high performance liquid chromatography. Dotted lines represent expected concentrations of the vitamin based on the contributions from the basal media formulation consisting of 40% DMEM and 40% F10. Data points represent the mean of three biological replicates ± standard deviation. Note that the *y*-axis scales are not all the same. cMFB primary embryonic chicken muscle fibroblasts. cMPC primary embryonic chicken muscle precursor cells. C2C12 murine myoblast-like cell line.

**Fig. 4:**
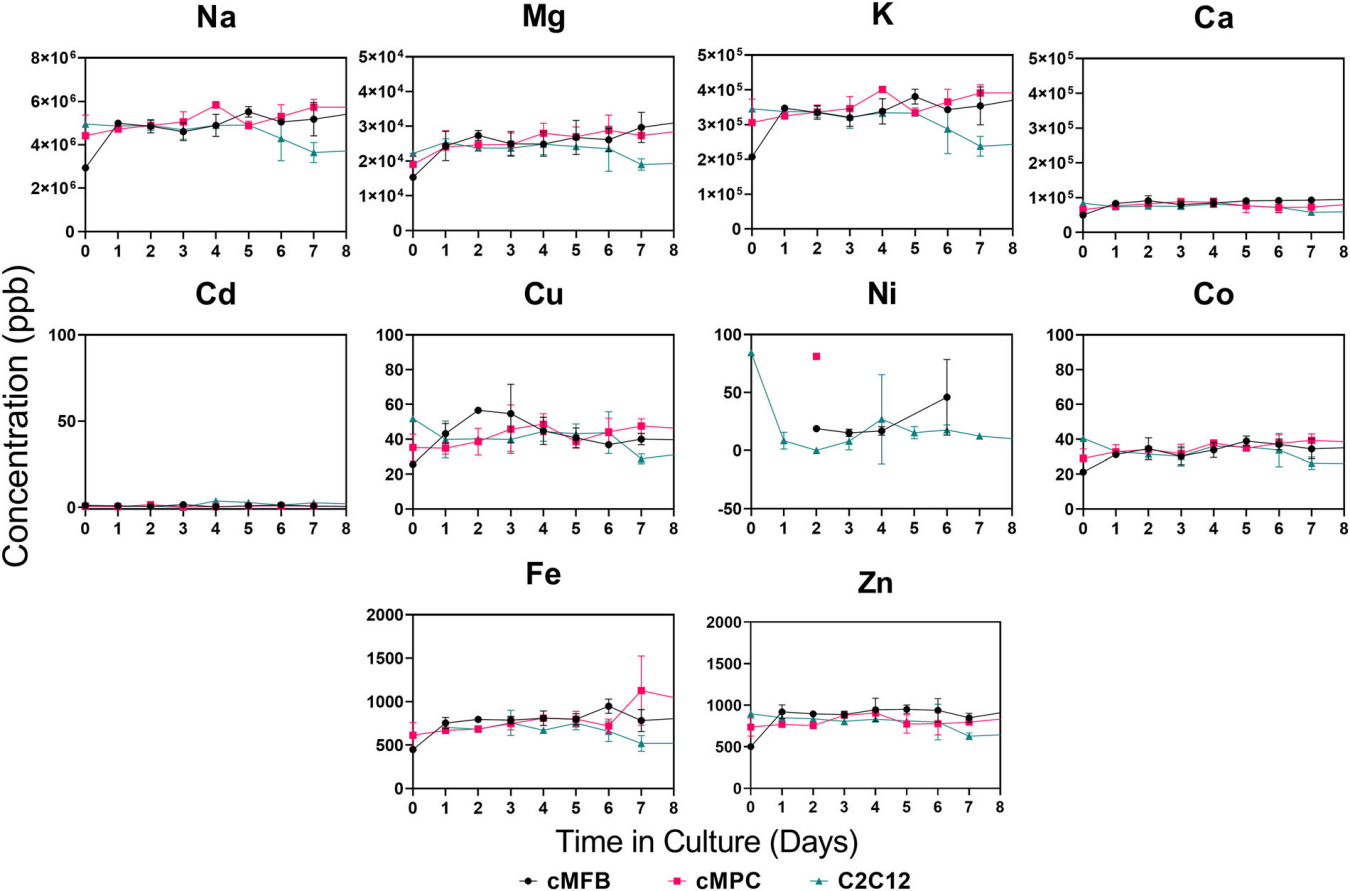
Minerals. Elemental analysis of spent media from cultures of three cultivated meat-relevant cell types, using inductively coupled plasma-mass spectrometry. Data points represent the mean of three biological replicates ± standard deviation. The *y*-axis scales vary widely between the graphs, so the figure does not directly indicate relative abundances of the different elements in the spent media samples. Several other elements were targeted for analysis but their concentrations in the samples were too low for detection. cMFB primary embryonic chicken muscle fibroblasts. cMPC primary embryonic chicken muscle precursor cells. C2C12 murine myoblast-like cell line.

As seen in Fig. 3, there is no appreciable decrease in the concentration of any of the water-soluble vitamins over time. Variations in the starting vitamin concentrations between the three cell types could potentially be explained by lot variation in the FBS that was used in the media, or by vitamin degradation in the samples during the handling and storage before analysis. Our recent review on cultivated meat media discusses these concerns in more detail^8^. Similarly, Fig. 4 displays no significant decrease in the elemental mineral concentrations measured by inductively coupled plasma-mass spectrometry (ICP-MS), nor any major difference in the concentrations between the three cell types. Additional elements analyzed included beryllium, aluminum, sulfur, vanadium, chromium, manganese, arsenic, selenium, strontium, molybdenum, silver, tin, antimony, barium, mercury, titanium, and lead; however, these elements were not present in the media at concentrations detectable by our ICP-MS methods (data not shown).

### Screening of cytokines and growth factors

**Fig. 5:**
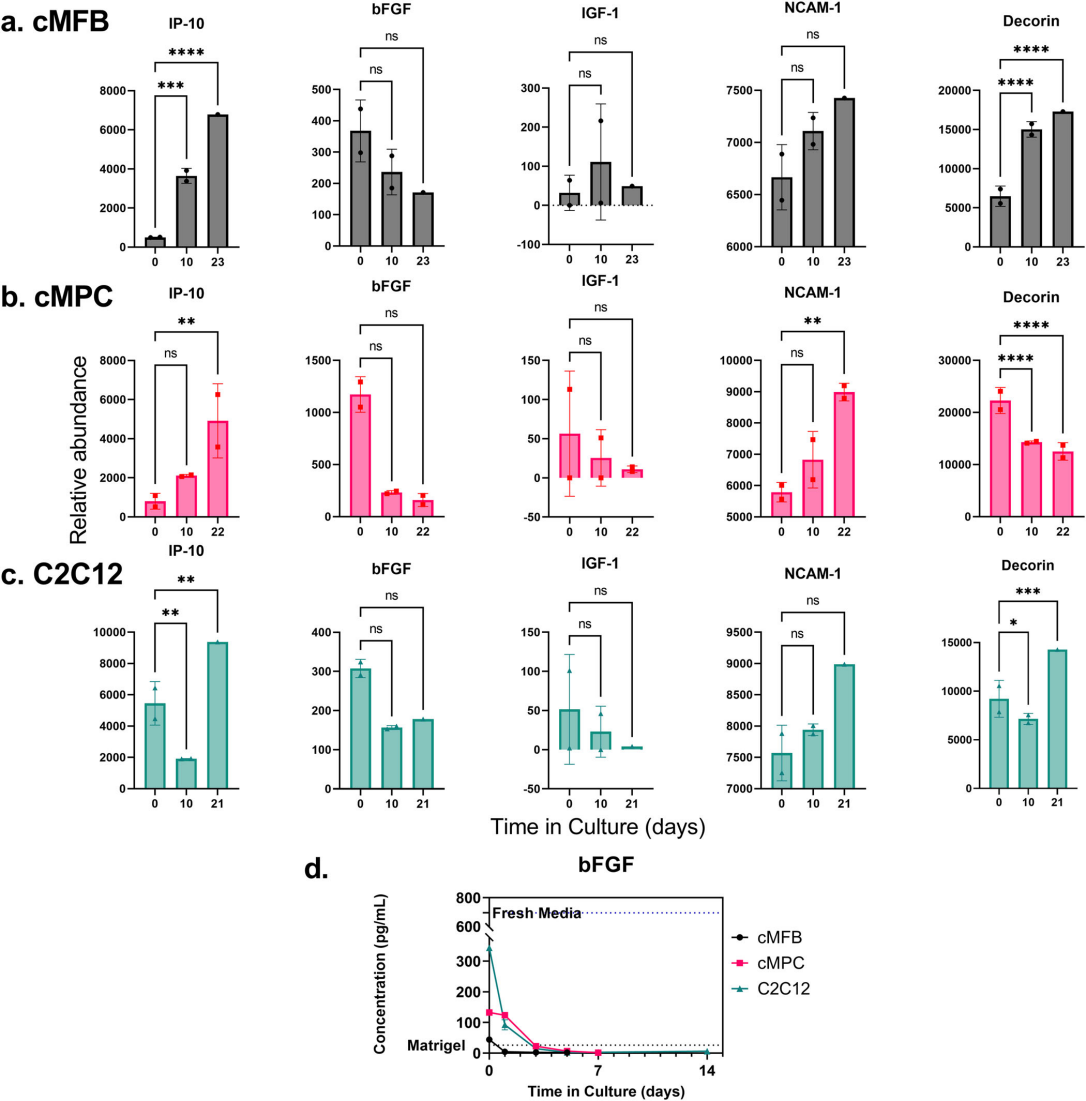
Cytokines + bFGF. Growth factor analysis of spent media from cultures of three cultivated meat-relevant cell types. A preliminary screening of 30 bovine cytokines was performed using a multiplex enzyme-linked immunosorbent assay (ELISA) to compare the concentrations in the spent media samples at three time points over 3 weeks. The five cytokines with the most significant changes over time were interferon gamma-induced protein 10 (IP-10), basic fibroblast growth factor (bFGF), insulin-like growth factor 1 (IGF-10), neural cell adhesion molecule 1 (NCAM-1), and decorin. The data for these proteins are plotted for the samples from (**a**) primary embryonic chicken muscle fibroblasts (cMFB), **b** primary embryonic chicken muscle precursor cells (cMPC), and **c** the C2C12 murine myoblast-like cell line. Data points represent the mean of two biological replicates ± standard deviation. Statistical analysis for this preliminary screening was performed using two-way analysis of variance (ANOVA) followed by the Holm-Šídák post-test for multiple comparisons with *α* = 0.05 (ns, not significant. *p < 0.05. **p < 0.01. ****p < 0.0001). It should be noted that statistical power was limited by having up to only two biological replicates per time point, but overall trends can be discerned in many cases despite lack of statistical significance. **d** Further analysis of basic fibroblast growth factor (bFGF) was performed using a standard ELISA and concentrations are shown with each cell type overlaid. Dotted lines represent the measured concentrations of bFGF in fresh medium that was prepared immediately before analysis as well as in Matrigel stock solution.

The initial screening of cytokine concentrations (Fig. 5a–c) did not reveal a significant difference in their depletion rates between the three cell types. It is important to bear in mind that only two biological replicates were able to be measured per cell type per time point, so the experiment was statistically underpowered. However, significant differences were observed in a few cases, such as with basic fibroblast growth factor (FGF2, bFGF), interferon γ-induced protein 10 (IP-10), neural cell adhesion molecule 1 (NCAM-1), and decorin. FGF2, the most relevant of these to CM media formulation, displayed a general decreasing trend over time in all three cell types, although the decrease was statistically significant for the cMPCs only. IP-10 and NCAM-1, in general, increased over time in all three cell types, whereas decorin decreased for cMPC, increased for cMFB, and decreased then increased for C2C12.

Due to the general decrease in FGF2 observed over time in all three cell types, and due to its known importance in the promotion of myoblast proliferation and inhibition of differentiation, we performed a more focused analysis on the changes in FGF2 over time in the spent media samples. This analysis included three biological replicates per time point tested per cell type, but we only focused on key time points due to assay sample number limitations. Figure 5d shows the overall relative FGF2 concentrations in the media over time between the three cell types, as well as the observed concentrations in freshly prepared medium and Matrigel working solution for reference. All three cell types displayed a marked exponential decrease in FGF2 until around 5 days, which generally corresponded with the increase in cell number. However, the cMFB culture tended to deplete FGF2 more rapidly, while its cell proliferation rate did not seem to be significantly influenced by the roughly 10-fold reduction in the concentration of the growth factor.

### Determination of specific utilization rates for key nutrients

**Fig. 6:**
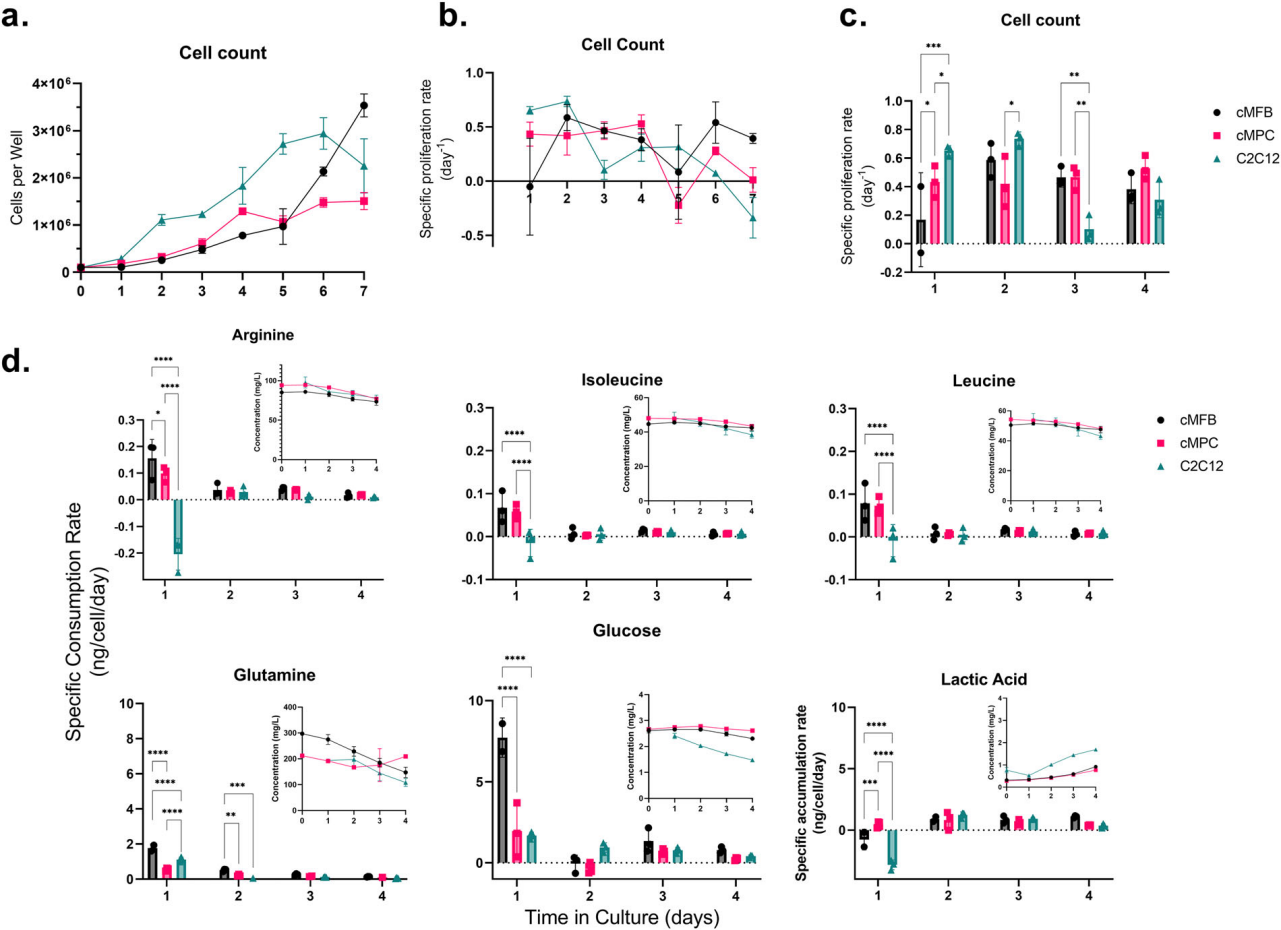
Specific growth and utilization rates. The specific cellular growth and nutrient utilization rates were calculated based on the spent media analysis data by finding the ratio of the approximate backward numerical derivatives for the cell count and nutrient concentration data to the cell count at the corresponding time point. **a** The total cell counts per well for the three cell types are plotted on a linear scale as a frame of reference. **b** The specific cellular proliferation rates are plotted as a line graph with the three cell types overlaid for the first 7 days of culture. **c** The same specific cellular proliferation rate data are plotted as a bar graph for the first 4 days of culture, allowing for visualization of the statistical differences between the three cell types. **d** The specific consumption rates of five key nutrients are plotted separately in bar graphs to compare the three different cell types. The specific accumulation rate of lactic acid is also included. Arginine, isoleucine, and leucine are plotted on *y*-axis scales up to 0.3 ng/cell/day, while glutamine, glucose, and lactic acid are plotted with scales ranging up to 10 ng/cell/day. Each bar graph includes an overlaid line graph showing the measured concentrations of the corresponding media component in mg/L as a reference. All error bars represent standard deviation. Statistical analysis for the specific proliferation rate and specific consumption rate data was performed using two-way analysis of variance (ANOVA) followed by the Holm-Šídák post-test for multiple comparisons with *α* = 0.05 (ns, not significant. *p < 0.05. **p < 0.01. ****p < 0.0001).

To obtain a better understanding of the potential differences between the nutrient utilization behaviors of the three cell types on a cellular basis rather than on a collective culture basis, we determined the specific utilization rates of the key nutrients that were identified in our spent media analyses. This analysis helped us to determine whether the utilization differences were due solely to the relative cell concentration or were a function of the differences in metabolic activities of individual cells. In this regard, Fig. 6a–c are intended to provide a better picture of the differences in cell proliferation between the cell types over the course of their exponential growth phase (roughly the first 4 days). Figure 6a presents the total cell count per well on a linear scale for the first 7 days of culture, while Fig. 6b shows the calculated specific proliferation rates in terms of cells produced per existing cell per day. These calculations are backward approximations of the derivatives using data between days 0 and 10 of culture. Figure 6c is a different representation of the same data shown in 6b and indicates that there are statistically significant differences between the three cell types which are dependent on the time in culture. Figure 6d includes the calculated specific utilization rates of glucose, glutamine, arginine, isoleucine, and leucine, which were the components that were found to be the most significantly depleted by the cells over time among all the media components we analyzed. The figure also includes the calculated specific production rates of lactic acid. Overall, Fig. 6d indicates that these calculated rates were significantly different between the three cell types only during the first day of culture (as well as at day 2 for glutamine). This finding highlights the more fundamental metabolic differences in the cells themselves and indicates that the variations in nutrient utilization are not simply due to differences in total cell numbers.

## Discussion

One of the exciting aspects of CM is the potential to allow for the mass production of a greater diversity of types of meat than is typically eaten by humans today. Whether due to cultural taboos or technical impracticalities, there are many species of animals that are not widely exploited for food. Nevertheless, even in conventional cuisines, there is still a considerable variety of meat that is commonly consumed. Therefore, the CM industry will almost certainly be tasked with producing many different types of meat at large scales if it hopes to displace some amount of conventional meat production and help satisfy the growing global population’s demand for nutritious and delicious dietary protein.

In the present paper, we present a straightforward characterization and comparison of three different cell types that are highly relevant to CM research and development. Murine C2C12s are not necessarily going to be useful in developing larger-scale CM bioprocesses in the longer term as the industry develops, although they are a useful model for bench-scale, early-stage CM research; indeed, they are used in many contemporary publications in the space^4,19–22^. Regardless, C2C12s, cMPCs, and cMFBs will not be the only cell types utilized in the CM industry, and the specific cellular behaviors that we observed in our study may not be generalizable to all other CM cell types. Moreover, the different bioprocesses and culture methods that could be used for CM will likely result in differing cell behaviors and nutrient requirements. Therefore, the aim of the present study is not to create a generalizable model for the growth characteristics of certain cell types, but rather to serve as a proof of concept that CM will require targeted media formulation and optimization efforts for each cell type, species, and bioprocess used for production. Further, the results suggest that several components found in conventional cell culture media may not be necessary to include in CM media, which could lead to significant cost savings, although more research needs to be performed in this regard.

The C2C12s we used in this study were seeded at passage 17, which is relatively high, especially compared to the primary chicken cells. C2C12s are an “immortalized” cell line: they are commonly used in studies at passage numbers upwards of 20^23–25^. We observed extensive differentiation of our C2C12s which occurred to roughly the same extent as the cMPCs, suggesting that the two cultures could be comparable in this regard despite the difference in their passage numbers. However, as indicated in Fig. 1c, the C2C12s began differentiating later (day 5) and at a higher cell density than the cMPCs (day 3), which could be due in part to the passage number difference, although it is more likely due to inherent physiological differences between the cell types themselves. The findings here in regards to passage number effects are not conclusive; it is certainly reasonable to assume that passage number will affect the metabolic activities and media nutrient requirements of cell populations. In fact, this subject has been extensively researched and proven over the years in many contexts, where studies generally demonstrate lower proliferation speeds, altered function, and different gene expression and phenotype patterns at higher passage numbers^24,26,27^. While the present study does not endeavor to reveal the effects of senescence and passage number on media requirements for cultivated meat, this is currently an active topic of research in our group because these will be important considerations for the large-scale proliferation of cells grown for CM over several doublings^8^.

The complex and undefined nature of FBS is an inherent challenge in laboratory-scale CM research^28^. FBS batch variability likely had an effect on the variation in the concentrations of specific signaling molecules and trace nutrients, in addition to the general growth behavior that we observed between the three cell types we tested^29^. The unpredictability of a scaled culture system using FBS would be a reason to prefer a chemically defined medium. However, using undefined and complex media ingredients derived from plants and agricultural waste streams is likely going to be important for producing CM at a competitive price compared to conventional meat^8^. Therefore, assessing the nutrient utilization rates of CM-relevant cell types grown in an FBS-containing medium can be justified here for the sake of understanding the major nutrient consumption trends that are observable in a complex medium.

Free amino acids have a variety of complex functions in cells, ranging from being the structural building blocks of proteins to participating in various metabolic and signaling pathways^30,31^. For instance, the interplay of serine and glutamate is implicated in the tetrahydrofolate pathway, influencing proliferation in a cell type-dependent fashion^32^. Based on the current understanding of amino acid metabolism, it is safe to assume that most amino acids will be required in CM cell culture media, even if they are not depleted by the cells over time. It is therefore challenging to predict and understand the effects of specific amino acid concentrations on the behaviors of all CM-relevant cell types, and more directed studies would need to be performed to elucidate these unknowns. However, based on our SMA, it is reasonable to imagine that primary cells should display a lower cellular metabolic rate than an immortalized cell line (like C2C12) that was selected for its ability to rapidly proliferate over many generations. This idea is reflected in the data shown in Fig. 6 for the first one or two days of the culture period. The figure also indicates that fibroblasts may not attain their highest specific growth rates as quickly as muscle cells after seeding. Further, the processes of cell differentiation, fusion, and hypertrophy in muscle cells and collagen production in cMFBs starting around days 5–7 complicate the understanding of per-cell utilization patterns. As the industry attempts to generate immortalized and optimized CM-relevant cell lines, it will be important to consider and investigate the effects of genetic manipulation as well as differentiation state on cell metabolism and media requirements.

Through our analysis of carbohydrates, amino acids, water-soluble vitamins, minerals, and cytokine proteins in our spent media samples, we found that only a select few of these components were appreciably decreasing in concentration over the cell culture period. These compounds included glucose, arginine, glutamine, isoleucine, leucine, methionine, serine, and FGF2. Decorin also displayed notable decreases and/or increases over time in the three cell culture groups. As a myokine proteoglycan that has been implicated in the regulation of both myogenesis and collagen synthesis, decorin likely plays a complex role in this context^33,34^. While decorin has not been extensively studied in the literature, it has been shown that it is secreted by contracting muscle cells^33^. The patterns of decorin abundance in our spent media samples could therefore be reflecting certain aspects of the overall health, differentiation status, and function of our cells, but this information itself is not enough to draw specific conclusions and ultimately may not be important in the context of cultivated meat media development.

The fact that the cMPC cultures overall did not appear to deplete glucose and potentially metabolized the glucose more completely (less lactate production) when compared with C2C12s and the cMFBs is intriguing. This may be due in part to the fact that the cMPCs were smaller in size compared to C2C12s and did not reach the same cell density as the cMFBs at confluence; it may also point to more fundamental differences in the metabolic activities between the cells. The dissimilar differentiation behavior between the cMPCs and C2C12s, which did not appear to be influenced or explained by glucose or FGF2 concentrations, also suggests there are more intrinsic physiological differences between the cell types. It is also worth noting the possibility of the cell density itself to influence the metabolic activity of the cells due to cell–cell interactions^35^, and this should be investigated further in larger scale suspension culture systems for cultivated meat.

The specific rates of nutrient consumption in Fig. 6d also clearly indicate that the cMFBs were using glucose and glutamine at a faster rate and producing less lactate at the beginning of the culture period compared to the other cell types. This suggests that the cMFBs may use glucose more completely (mitochondrial oxidative phosphorylation), whereas the cMPCs use glucose more in glycolysis and release lactate rather than completely metabolizing pyruvate in the mitochondria. It could be useful for future work to investigate the underlying cellular mechanisms that lead to these differential glucose and amino acid utilization rates. The main conclusion that can be drawn from Fig. 6d, however, is that at the early phase of cell proliferation the specific cell lineage and species origin have a significant influence on the cellular rates of utilization of key media nutrients, and these differences may be less significant later in the cell culture period. This understanding could facilitate optimization for feed rates in batch fed bioprocesses that may be used in cultivated meat production.

It is already well-understood that many components of cell culture media are inherently unstable in solution^30,36,37^. It is certainly possible that chemical degradation played a major part in the concentration changes we observed in our spent media samples over time. However, since our study was intended simply to reveal whether component utilization rates vary between CM-relevant cell types, and not to create models for how these cells use the nutrients, the results reported here can still be considered valid in context of constant intrinsic rates of component degradation independent of cell metabolic activity. Also, in practice for CM production, cells would not likely be left in the same media for more than 5–7 days at maximum, suggesting that media instability would be even less of a concern.

In the present study, we confirm a foundational tenet of cultivated meat media formulation: the specific nutrient requirements of different CM-relevant cell types cannot be assumed to be similar. Future studies should examine whether chemically defined or complex serum free media formulations will also be so dependent on species and cell type, as well as how these media affect cellular metabolism compared to serum-containing media. It would also be interesting to investigate whether supplying excessive amounts of various nutrients could negatively affect cell growth. As the industry continues to develop, there will likely be an increasing need for more robust, streamlined, and accessible media optimization techniques^9^. If academic and industry researchers can rise to meet this demand, the path toward bringing CM products to market will become substantially clearer.

## Methods

### Chicken cell isolation and storage

We developed and tested a method based on existing published procedures^38–41^ and empirical observations to isolate cMPC from 19-day-old Hy-Line chicken embryos in accordance with the relevant policies and guidelines of the Institutional Animal Care and Use Committee of the University of California, Davis. Briefly, fertile eggs were sanitized with 70% ethanol before opening and euthanizing the embryos via decapitation. Pectoral muscles were extracted and placed in 20–30 mL sterile phosphate buffered saline (PBS) with 1x penicillin, streptomycin, and amphotericin B solution. Enzymatic and mechanical tissue dissociation was performed using a gentleMACS skeletal muscle tissue dissociation kit (Miltenyi Biotec, Auburn, CA). A short pre-plating step of 25 min on uncoated plastic culture flasks was used to remove many of the rapidly adhering non-MPC cell types in order to enrich the MPC population of the primary cell culture. Cells were seeded at a density of 17,000 cells per cm^2^ of Matrigel-coated surface area (Corning, Corning, NY) during the pre-experiment expansion phase. The cells’ ability to readily differentiate into well-developed myotubes after reaching confluence confirmed their identity as muscle precursors. cMFBs were derived by culturing a subset of the obtained cells on uncoated TC-treated plastic, which selected for the fibroblastic cell types that could better adhere to the plastic surface and synthesize their own extracellular matrix. The cMFBs and cMPCs were passaged (or media were changed) every two to 3 days during initial expansion. They were allowed to expand as much as possible from passage 0 through 1 without allowing surfaces to become more than about 70% confluent. After the first passage, all cells were collected and frozen at 3.5 × 10^6^ cells per 1 mL aliquot of freezing medium (70% DMEM, 20% fetal bovine serum, 10% dimethyl sulfoxide) and stored in liquid nitrogen using conventional techniques.

### General cell culture

The three types of cells (cMFB, cMPC, and C2C12) were each expanded in the experimental medium to generate enough cells to set up the SMA experiment (at least 10^7^ cells). This medium consisted of 40% DMEM (containing high glucose, pyruvate, and l-glutamine), 40% Ham’s F10 nutrient mix, 20% fetal bovine serum (FBS), and an additional 2.5 ng/mL of recombinant human basic fibroblast growth factor (hFGF2) (Cell Signaling Technology, Danvers, MA). The basal media and FBS were all obtained from Gibco (ThermoFisher Scientific, Waltham, MA). During the pre-experiment cell culture phase, the cells were grown on Matrigel-coated 15 cm polystyrene Petri dishes. During passaging, they were dissociated using TrypLE Express reagent (Gibco) and counted using a hemocytometer with trypan blue. Cells were kept in humidified incubators at 37 °C and 5% CO_2_.

### Experimental media collection phase

The three cell types were seeded onto Matrigel-coated 6-well plates at 10^5^ cells per well. cMFBs and cMPCs were seeded at passage 3, while C2C12s were seeded at passage 17. Thirty wells were seeded per cell type to allow for a parallel culture setup for multiple collection time points in triplicate and to yield sufficient statistical power. All the spent media were collected from three wells per cell type at each of the predetermined time points, while the remaining wells were left to continue culturing, without media changes, until their planned collection time. The collected spent media volumes were recorded (to account for evaporation during culture), filtered through 0.22 µm membrane filters, and stored at −30 °C for later analysis. Directly after media collection at each time point, the cells remaining in the wells were imaged using an ImageXpress Pico automated microscope (Molecular Devices, San Jose, CA), dissociated, and manually counted via hemocytometer.

### Glucose and lactic acid analysis

An Agilent 1260 Infinity II high performance liquid chromatography (HPLC) system (Agilent Technologies, Santa Clara, CA) coupled to a G7162A refractive index (RI) detector was used with an Aminex HPX-87H column (Bio-Rad Laboratories, Hercules, CA) and a conventional method for measuring carbohydrates and organic acids according to the manufacturer’s suggestions. The mobile phase was 5 mM sulfuric acid and the HPLC was operated at an isocratic 0.6 mL/min flow rate with the column kept at 50 °C. Samples were centrifuged at 10,000 × g for 10 mins and filtered through 0.22 µm polyvinylidene difluoride membrane filters before injection. Injection volume was 20 µL and run time was 20 min. Standards of D-( + )-glucose and sodium L-lactate were obtained from Sigma-Aldrich (St. Louis, MO) and were prepared fresh prior to analysis to generate standard calibration curves with five points that captured the range of sample concentrations.

### Amino acid analysis

Amino acid analysis was performed by the UC Davis Molecular Structure Facility using a Hitachi L-8900 amino acid analyzer (Hitachi High Tech, Tokyo, Japan). Samples were acidified to 2% sulfosalicylic acid (SSA) and incubated at room temperature (23 °C) for 15 min before being frozen (−20 °C) overnight. They were then diluted with 100 nmol/mL AE-Cys Li. 20 µL of sample was injected into the system, and free amino acids were separated using ion-exchange chromatography with a post-column ninhydrin reaction. Column and buffers were obtained from Hitachi, ninhydrin was supplied by Wako (Richmond, VA), and amino acid standards were obtained from Sigma-Aldrich (St. Louis, MO). Absorbance was recorded at both 570 nm and 440 nm after the reaction with ninhydrin to determine the response factor for each individual amino acid and to quantify levels relative to the known amino acid standards. AE-Cys was used to correct for injection volume variance. Certain amino acids such as tryptophan could not be measured using this method.

### Vitamin analysis

To measure water-soluble vitamins, an Agilent 1200 Infinity HPLC system coupled to a diode array detector was used with an Agilent Poroshell 120 EC-C18 column at 4.6 ×100 mm with particle size of 2.7 µm. The HPLC method was developed by the UC Davis Department of Viticulture and Enology analytical laboratory based on manufacturer suggestions. Briefly, mobile phases consisted of 25 mM HK_2_PO_4_ (pH 7.0) (A) and acetonitrile (B). The 20.1 min run time was set up with a constant flow rate of 0.5 mL/min. The flow consisted of 1% B at 0 to 5 mins, a gradient increase to 30% B from 5 to 15 mins, 30% B from 15 to 20 mins, a gradient decrease to 1% B from 20 to 20.1 mins, followed by 5 mins post run time at 1% B. Data were acquired at 205, 214, 232, 266, and 280 nm. The column was kept at 35 °C, and injection volume was 20 µL. Vitamin standards were obtained from Sigma and were prepared fresh and shielded from light prior to analysis.

### Mineral analysis

A 7850 inductively coupled plasma-mass spectrometer (ICP-MS) (Agilent Technologies, Santa Clara CA), which includes the Octopole Reaction System ORS^4^ collision cell and Ultra High Matrix Introduction (UHMI) aerosol dilution system, was used for the analysis. Sampling was performed using an SPS 4 autosampler (Agilent Technologies). The ICP-MS was configured with the standard sample introduction system consisting of a MicroMist glass concentric nebulizer, temperature-controlled quartz spray chamber, and quartz torch with a 2.5 mm id injector. The interface consisted of a nickel-plated copper sampling cone and nickel skimmer cones.

ICP-MS uses an (ORS^4^) cell operating in helium (He) collision cell mode with Kinetic Energy Discrimination (KED). This combination provides the optimum configuration to control common polyatomic interferences. This ICP-MS has a wide linear dynamic range (10 or 11 orders of magnitude), so major and trace elements in spent media samples were measured in a single run. The calibration standards were prepared in 2% nitric acid (HNO3) and 0.5% hydrochloric acid (HCl). HCl is routinely added to samples for analysis, as it ensures that chemically unstable elements such as Hg are retained in the solution. The Calibration standards were prepared from environmental calibration standard, p/n 5183-4688 (Agilent Technologies), and 1000 µg/mL single calibration standard for Hg, p/n 5190-8485 (Agilent Technologies). Most elements were calibrated from 0.1 to 100 ppb. Major Minerals were calibrated from 10 to 10000 ppb. Hg was calibrated from 0.01 to 2.0 ppb. National Institute of Standards and Technology (NIST, Gaithersburg, US) SRM 1643f was used to check the ICP-MS calibration as an Initial calibration verification (ICV). Continuing calibration verification (CCV) standards were prepared at the midpoint of the curve (most elements 50 ppb, majors 5000 ppb, and Hg 1ppb).

The internal standard (ISTD) solution containing 2 ppm Sc, Ge, Rh, In, Bi, and Lu, was prepared in 2% HNO3, 0.5% HCl, and 10% isopropanol (IPA). The ISTD solution was automatically added at a flow rate ~16 times lower than the sample flow.

### Growth factor analysis

A panel of 30 bovine cytokines was assessed using a multiplex enzyme-linked immunosorbent assay (ELISA) (Cat.# GSB-CAA-30, RayBiotech, Peachtree Corners, GA). Only a subset of the collected spent media samples and replicates was analyzed using this array due to sample number and volume limitations. This analysis served to provide a general indication of the relative abundance of these proteins in the samples and whether they may be changing significantly in the media over the culture period. Bovine FGF2 was then measured more precisely in more samples with a standard sandwich ELISA kit according to manufacturer instructions (ThermoFisher Cat.# EB2RB), using a SpectraMax iD3 multimode plate reader to measure absorbance at 450 nm (Molecular Devices, San Jose, CA).

### Data and statistical analysis

Data obtained from the various analytical techniques were converted into mass per volume ratios and plotted versus the day of cell culture. For the multiplex cytokine screen, statistical analysis was performed using two-way analysis of variance (ANOVA) followed by the Holm-Šídák post-test for multiple comparisons where α = 0.05. For the calculation of the specific cellular rates of nutrient utilization, numerical derivatives were obtained between the concentration values at a given time point and the point preceding it. These backward derivative approximation values were divided by the cell numbers at each respective time point to give specific utilization or production rates and plotted versus time in culture. Statistical significance was assessed using two-way ANOVA with the Holm-Šídák post-test for multiple comparisons where α = 0.05. Prism 9 (GraphPad Software, San Diego, CA) was used to create all charts and perform the statistical analyses.

## Supplementary information

Supplemental Figure 1

## Data Availability

The authors declare that all data supporting the findings of this study are available in the paper and supplementary information.
