# Supplementary material for: Spent media analysis suggests cultivated meat media will require species and cell type optimization
Source: NPJ Sci Food. 2022 Sep 29;6:46. doi: 10.1038/s41538-022-00157-z (PMC9523075; doi:10.1038/s41538-022-00157-z)
Supplement: Supplementary file 1 — Supplemental Figure 1 [file 41538_2022_157_MOESM1_ESM.pdf]

Supplementary Figures

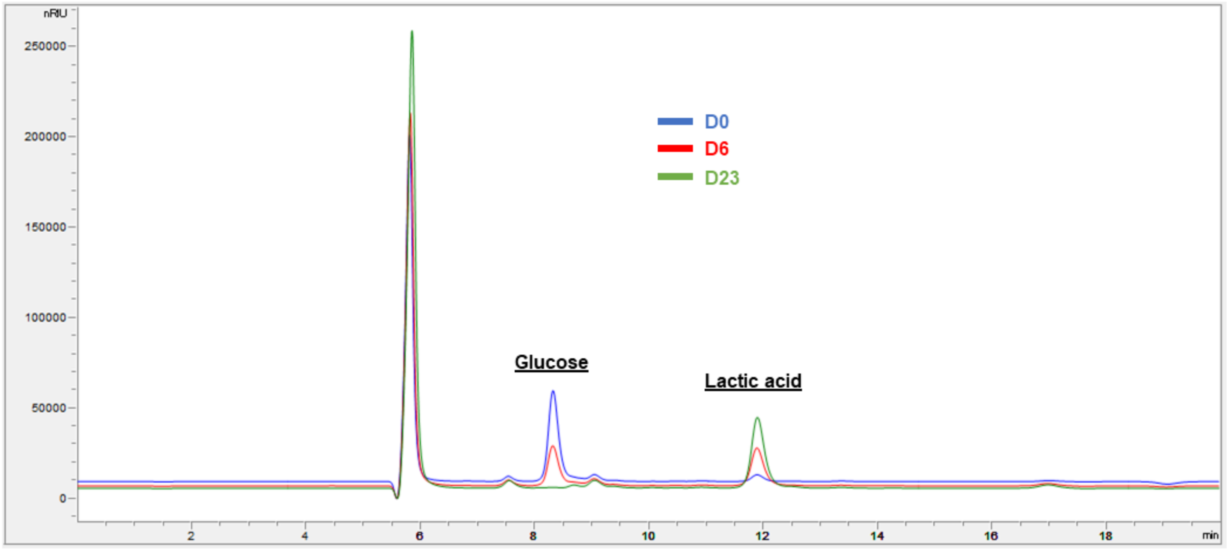

Supplementary figure 1: Chromatogram from glucose and lactate analysis

An overlaid example of three chromatograms obtained from the HPLC method used to measure glucose and lactate in our spent media samples. The three chromatograms are all from the C2C12 group, and represent media samples taken at day 0, 6, and 23 of culture according to the figure legend. The only peaks that significantly varied across the time point samples were those known to correspond to glucose and lactate. The other peaks visible in the chromatograms were more substantially similar across samples, and represent other components of the sample matrices that were not changing in concentration over the culture time period.
